# Correction: A novel water-from-air technology: creeping clathrate desalination of deliquescent salt solutions

**DOI:** 10.1039/d5ew90044a

**Published:** 2025-12-09

**Authors:** Anke Snauwaert, Estelle Becquevort, Maarten Houlleberghs, Robin Peeters, Sambhu Radhakrishnan, Eric Breynaert, Johan Martens

**Affiliations:** a Centre for Surface Chemistry and Catalysis Characterisation and Application Team (COK-KAT), KU Leuven Leuven 3001 Belgium johan.martens@kuleuven.be; b NMRCoRe-NMR/X-Ray platform for Convergence Research, KU Leuven Leuven 3001 Belgium; c Center for Molecular Water Science (CMWS) Notkestraße 85 22607 Hamburg Germany

## Abstract

Correction for ‘A novel water-from-air technology: creeping clathrate desalination of deliquescent salt solutions’ by Anke Snauwaert *et al.*, *Environ. Sci.: Water Res. Technol.*, 2025, **11**, 2926–2934, https://doi.org/10.1039/D5EW00838G.

The authors regret the omission of one of the authors, Robin Peeters, from the original manuscript. The correct list of authors and affiliations for this paper is as shown above. The author contribution section should read “Investigation: A. S., M. H. and R. P.; formal analysis: A. S. and M. H.; writing – original draft: A. S.; validation and visualization: A. S., E. Be. and S. R.; supervision: E. Br. and J. M.; conceptualization: A. S., M. H., R. P., E. Br. and J. M.; funding acquisition: E. Br. and J. M.; review and editing: S. R., E. Br. and J. M.”.

The Royal Society of Chemistry apologises for these errors and any consequent inconvenience to authors and readers.

